# Interaction between Gut Microbiota and Curcumin: A New Key of Understanding for the Health Effects of Curcumin

**DOI:** 10.3390/nu12092499

**Published:** 2020-08-19

**Authors:** Beatrice Scazzocchio, Luisa Minghetti, Massimo D’Archivio

**Affiliations:** 1 Center for Gender-Specific Medicine, Istituto Superiore di Sanità, 00161 Rome, Italy; beatrice.scazzocchio@iss.it; 2 Research Coordination and Support Service, Istituto Superiore di Sanità, 00161 Rome, Italy; luisa.minghetti@iss.it

**Keywords:** gut microbiota, curcumin, polyphenols, health

## Abstract

Curcumin, a lipophilic polyphenol contained in the rhizome of *Curcuma longa* (turmeric), has been used for centuries in traditional Asian medicine, and nowadays it is widely used in food as dietary spice worldwide. It has received considerable attention for its pharmacological activities, which appear to act primarily through anti-inflammatory and antioxidant mechanisms. For this reason, it has been proposed as a tool for the management of many diseases, among which are gastrointestinal and neurological diseases, diabetes, and several types of cancer. However, the pharmacology of curcumin remains to be elucidated; indeed, a discrepancy exists between the well-documented in vitro and in vivo activities of curcumin and its poor bioavailability and chemical instability that should limit any therapeutic effect. Recently, it has been hypothesized that curcumin could exert direct regulative effects primarily in the gastrointestinal tract, where high concentrations of this polyphenol have been detected after oral administration. Consequently, it might be hypothesized that curcumin directly exerts its regulatory effects on the gut microbiota, thus explaining the paradox between its low systemic bioavailability and its wide pharmacological activities. It is well known that the microbiota has several important roles in human physiology, and its composition can be influenced by a multitude of environmental and lifestyle factors. Accordingly, any perturbations in gut microbiome profile or dysbiosis can have a key role in human disease progression. Interestingly, curcumin and its metabolites have been shown to influence the microbiota. It is worth noting that from the interaction between curcumin and microbiota two different phenomena arise: the regulation of intestinal microflora by curcumin and the biotransformation of curcumin by gut microbiota, both of them potentially crucial for curcumin activity. This review summarizes the most recent studies on this topic, highlighting the strong connection between curcumin and gut microbiota, with the final aim of adding new insight into the potential mechanisms by which curcumin exerts its effects.

## 1. Introduction

Curcumin, one of the major curcuminoids contained in the rhizome of *Curcuma longa* (turmeric), is a lipophilic polyphenol that has been used for centuries as an essential tool of traditional medicine in Asia [1]. Nowadays, it is widely used as dietary spice, but also in cosmetic and pharmaceutical industries [2].

Curcumin has received considerable attention in the last years for its pharmacological activities. Due to the presence of conjugated double bonds in its chemical structure, this polyphenol serves as an effective electron donor to counteract the production of reactive oxygen species (ROS) in many redox reactions [3], acting as a potent antioxidant. In addition, it has other important biological functions, such as anti-inflammatory, antitumor, antimicrobial, and antiviral ones [4,5,6,7].

Different studies highlighted that curcumin, like other dietary polyphenols, counteracts the effects of toxic damage in different tissues [8,9] and, in addition, it is able to interfere with key cancer-associated signaling pathways by directly targeting proteins or regulating gene expression [10,11]. According to its biological activities, curcumin has been proposed as a potential treatment for many diseases, among which are gastrointestinal, cardiovascular, and neurological disorders, diabetes, and several types of cancer [12,13].

Unfortunately, these findings have not been consistently supported through human clinical trials, except for the treatment of arthritis, pain, and major depressive disorder [14,15,16]. Consequently, the real biological activities of curcumin remain to be better elucidated; indeed, a discrepancy exists between the well-detailed in vitro and in vivo activities of curcumin and its poor bioavailability and chemical instability that should limit any healthy therapeutic outcome.

High concentrations of curcumin have been detected in the gastrointestinal tract after oral administration, and this has led to the hypothesis that the polyphenol directly exerts its regulatory effects on gut microbiota, explaining in this way the paradox between its low systemic bioavailability and its wide pharmacological activities that would be mediated by the gut microbiota.

It is well known that the gut microbiota has several important roles in normal human physiology, and its composition can be influenced by a multitude of environmental and lifestyle factors [17,18,19]. Accordingly, any perturbations in gut microbiome profile, that is, dysbiosis, can have a key role in human disease progression. Interestingly, curcumin and its metabolites have been shown to influence the gut microbiota [20,21]. It is worth noting that the interaction between curcumin and gut microbiota gives rise to two different phenomena: the first is the direct regulation of intestinal microflora by curcumin and the second is the biotransformation of curcumin by gut microbiota, yielding active metabolites [22,23]; both these phenomena seem to be crucial for the activity of curcumin.

This review summarizes the most recent studies on the reciprocal interaction between curcumin and gut microbiota, with the final aim to provide novel insight for defining future effective preventive strategies and microbiota-targeted therapies using curcumin. The observed high concentrations of curcumin in the GI tract after oral administration can lead to two major effects: an altered gut microbiota and the modulation of intestinal functions. We focused our literature search on the altered gut microbiota. The search was conducted in PubMed on 29 May, 2020. The search syntax was “curcumin”, “microbiota”, and “microbiome”. The scientific literatures were searched for in vivo, experimental and clinical studies, and human randomized controlled trials, reporting results on the interaction between curcumin and gut microbiota and vice versa.

The publication date was considered from 29 May, 2015 to 29 May, 2020 (five years).

In total, 89 titles were found through database search: after excluding review articles, studies in animal models and in humans were identified and discussed more in detail.

## 2. Curcumin: Metabolism and Bioavailability

Curcumin, [1,7-bis(4-hydroxy-3-methoxyphenyl)-1,6-heptadiene-3,5-dione], is the most representative polyphenol component extracted from the rhizome of *Curcuma longa* (turmeric). It is almost completely insoluble in water but it is easily soluble in organic solvents such as acetone and ethanol [24], and it is quite stable in the acidic pH of the stomach [25]. From a chemical viewpoint, the molecule is symmetric with two similar aromatic rings, and presents conjugate double bonds utilized as effective electron donor to hinder ROS formation.

Curcumin is widely consumed, particularly in Asia, as one of the culinary ingredients in food recipes. In the recent years, this polyphenol has increasingly received worldwide attention for its multiple pharmacological activities, primarily anti-inflammatory and antioxidant ones [26,27,28,29]. A recent meta-analysis has evidenced curcumin efficacy as a free radical scavenger and an inhibitor of malondialdehyde production, showing its ability in improving levels of antioxidants in diseased individuals susceptible to oxidative stress. The reduction of oxidative stress by curcumin supplementation was dependent on the dose and the duration of treatment [30].

Curcumin and the whole turmeric rhizome have some beneficial effects in the treatment of chronic diseases such as gastrointestinal, cardiovascular, and neurological disorders, diabetes, and several types of cancer [31,32,33,34,35]. Clinical trials based on curcumin administration have been published or are currently in progress, pointing out the expanding interest of the scientific community on the therapeutic potential of curcumin [36,37,38,39].

The safety of orally administered curcumin has been clearly demonstrated: the US Food and Drug Administration has approved curcumin as a compound “generally recognized as safe” and also JECFA (The Joint FAO/WHO Expert Committee on Food Additives) and EFSA (European Food Safety Authority) reported the ADI (acceptable daily intake) value of 0–3 mg·kg^−1^ for curcumin [40]. However, it has to been taken into account that very few reports on the potential adverse effects of curcumin exist: recently, a case report showed a liver injury attributed to a curcumin supplement in a woman with jaundice [41]. Curcumin could also interfere with systemic iron metabolism, suggesting limited application of this compound in patients with chronic disease or anemia [42].

In spite of its therapeutic potential against a wide spectrum of human pathologies, curcumin is known for its poor gastrointestinal absorption and low bioavailability, mainly attributed to water insolubility, and rapid metabolism and excretion [43].

**Figure 1:**
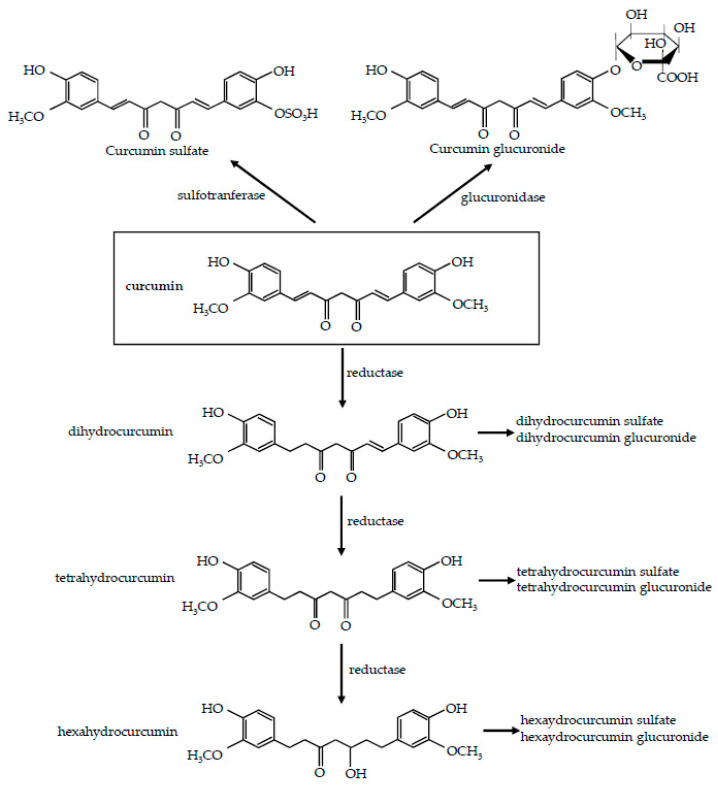
Metabolism of curcumin. Reduction pathway and the two main conjugation pathways, glucuronidation and sulfatation, are shown.

In humans, after curcumin oral administration, glucuronide conjugates and sulfate conjugates are detected in blood, while intact curcumin is poorly detected [44]. As a first step, ingested curcumin passes through the stomach, where practically no absorption takes place. Due to its resistance to low pH, curcumin, without any chemical modifications, reaches the large intestine and undergoes extensive phase I and II metabolism. Firstly, it is metabolized by phase I enzymes: different reductases introduce reactive and polar groups in their substrates, yielding active metabolites, namely, dihydrocurcumin, tetrahydrocurcumin, and hexahydrocurcumin [45,46,47] (Figure 1). This reductive metabolic reaction of curcumin occurs extensively in enterocytes and hepatocytes [46,48,49].

Then, these phase I metabolites undergo phase II metabolism: in vitro and in vivo study have previously demonstrated that curcumin and its reductive metabolites are easily conjugated [45,46,50]. Glucuronidases and sulfotrasferases are capable of conjugating glucuronic acid or sulphate molecule, respectively, to any of the hydroxyl groups [51], to produce the corresponding glucuronide and sulfate O-conjugated metabolites [22] (Figure 1). The conjugation process typically involves the addition of a single moiety, although double glucuronidation has been reported in isolated liver microsomes [52], and diglutathionylated curcumin has been found in isolated reaction systems [53]. The predominating pathway of conjugation is represented by glucuronidation; indeed the glucuronide of curcumin is usually found as the major metabolite of curcumin in body fluids, organs, and cells [47,54] even though, due to the increase of molecular weight, these metabolites are less active than their substrates [54,55].

The very low concentrations of curcumin in blood plasma and urine after oral administration have been demonstrated in both animal and human studies. It is important to underline that this could be due to the fact that curcumin derivatives are not always assayed, thus underestimating its absorption. In rats administered with an oral dose of 1000 mg/kg of curcumin, about 75% was excreted in feces, and a very low amount was detected in the urine [56]. An oral dose of 0.1 g/kg administered to mice yielded a peak plasma concentration of free curcumin that was only 2.25 mg/mL [47]. Even after high oral doses (up to 8 g/day), serum levels of curcumin were undetectable in humans [57,58]. In another clinical trial with an oral dose of 3.6 g of curcumin, a plasma level as low as 11.1 nmol/L was detected an hour after oral dosing [59].

This low bioavailability of curcumin after oral administration could largely restrict its pharmacological potential and consequently, its clinical application [60,61]. As a result, different delivery systems including micelles, liposomes, phospholipid complexes, microemulsions, nanostructured lipid carriers, and biopolymer nanoparticles have been developed to increase curcumin bioavailability. Specifically, Kato et al., by using a new formulation where curcumin was dispersed with colloidal nanoparticles, succeeded in improving hyperglycemia via stimulation of GLP-1 (glucagon-like peptide 1) secretion and the subsequent insulin secretion [62], suggesting a possible use of curcumin formulation in diabetes treatment. Such formulations may also be effective against inflammatory status and osteoarthritis [63,64], even if the dosage represents a critical point because it should remain quite low to avoid toxicity. Very recently, Chen et al. [65] clearly demonstrated that the supplementation of nanobubble curcumin extract in mice had a beneficial effect on health and exercise performance, helping mice to overcome physical fatigue. Moreover, several natural agents have been used to improve curcumin bioavailability, most of which work by blocking curcumin metabolism in order to increase its absorption [66]. Among these agents, piperine, the major active component of black pepper [67], probably represents the most utilized one [68,69,70].

Some recent papers have also showed the importance of food matrix in curcumin absorption [71,72], highlighting an enhanced bioavailability when it is consumed as fresh or powdered turmeric with respect to supplements, which could be due to the synergic activity with other turmeric compounds and/or to a turmeric matrix effect [71].

However, as previously stated, curcumin could exert its main regulative effects primarily in the gut, where high concentrations are present after oral administration [73]. Actually, curcumin is able to modulate directly intestinal barrier function as well as dysregulated signaling pathways. On the other hand, it might act at intestinal level by promoting changes in the composition and diversity of the gut microbiota [74]. The possible role of the gut microbiota in the mechanisms responsible for the biological activities of curcumin represents an interesting and attractive area of research and will be discussed in detail in the following paragraphs.

## 3. Gut Microbiota

There is considerable attention given to the substantial discrepancy between the strong biological effects of some functional foods and the poor bioavailability of these substances. For orally administered drugs and functional food, the bioavailability is defined as “the quantity or the proportion of the ingested dose that is directly absorbed in the small intestine to enter into circulation”. However, since the gut microbiota is actually considered as an effective bioreactor in the human intestinal tract and considering the emerging interactions between functional food and gut microbiota, probably we should think of a redefinition of the concept of bioavailability.

Recently, high concentrations of curcumin have been detected in the gastrointestinal tract after oral administration [20], thus suggesting that this polyphenol could directly interact with the gut microbiota exerting its regulatory effects. Before analyzing in more detail the mutual interaction between curcumin and gut microbiota, discussing the more recent findings on this topic, we will give a brief overview on human gut microbiota.

In the last decades, human microbiota has emerged as an area of utmost interest, as many studies have highlighted its impact on health and diseases [75]. It develops together with the host and fulfils essential physiological functions for the host, such as preventing infection, promoting the immune system maturation [76], participating in the regulation of nutritional absorption and metabolism [77], producing soluble B-vitamins (cobalamin, thiamine, pyridoxine, biotin, folate, nicotinic acid, pantothenic acid) and vitamin K lactic acid [78,79]. The colonization of newborn microbiota begins in utero [80] and changes suddenly during the first year of life.

The gut microbiota is a hugely complex ecology of organisms, primarily comprising many classes of bacteria (50 bacterial phyla and about a thousand of bacterial species), fungi, viruses, and a few other species [81,82]. Collectively, the whole of all microbiota genes, the microbiome, is 150 times larger than the human genome [83,84]. The gut microenvironment mainly favors the growth of bacteria from seven predominant phyla: Bacteroidetes, Firmicutes, Actinobacteria, Fusobacteria, Proteobacteria, Verrucomicrobia, and Cyanobacteria [85], with the first two phyla constituting more than 90% of the total gut population. Most of the species under Firmicutes, such as *Clostridium*, *Eubacterium*, and *Ruminococcus*, are the most representative in the gut [86].

The composition of microbiota varies greatly within each individual, in which about 150 bacterial species can predominate, getting benefits from the nutrient-rich environment of the gut and performing protective, metabolic, and structural functions. Understanding this variability in the “healthy microbiome” represents one of the major challenge in microbiota research.

The gut microbial community is very dynamic and has specific properties that allowed it to colonize the gut, among which are the possession of enzymes able to utilize the available nutrients, the right cell-surface molecular pattern to attach at the “right” habitat, and the ability to evade bacteriophages [86]. Usually, these microbes are mainly involved in nutrient metabolism through fermentation of complex carbohydrates. This leads to the synthesis of short-chain fatty acids, well known for their anti-inflammatory and anticancer properties, which represent an important energy source for colonocytes. Intestinal microorganisms might also influence lipid and protein metabolism [87].

There is growing evidence that any perturbation in gut microbiota composition (dysbiosis), associated with a reduced diversity and the predominance of a few pathogenic taxa, is closely linked to many human diseases [88,89,90]. In particular, dysbiosis has been related to pathological gastrointestinal conditions, such as inflammatory bowel disease and colorectal cancer (CRC) [91,92], but also to obesity, diabetes, asthma, and allergies [93,94].

Microbiota composition can be influenced by a multitude of environmental and lifestyle factors, among which dietary habits have a great impact on gut microbiome diversity. Del Bas et al. [95] have highlighted that the correct balance between fibers, simple carbohydrates, and fats is crucial in determining the abundance of different gut microbial populations. It has also been shown that unbalanced diets cause alterations in gut microbiota composition, resulting in modification of gut permeability and in gut low-grade inflammation [96].

In view of the above, it is understandable the increasing interest in defining the effective interplay between curcumin and gut microbiota.

## 4. Curcumin Modifies Gut Microbiota

As stated above, curcumin preferentially accumulates in the gastrointestinal tract after oral or intraperitoneal administration, and therefore it is reasonable to hypothesize that this polyphenol may exert its regulatory effect by modulating the microbial richness, diversity, and composition of the intestinal microflora [97]. Many in vivo studies have confirmed this hypothesis, and the most recent and interesting are discussed below.

A research has been carried out on adult healthy volunteers [98] asked to consume daily for 28 days a dried *Curcuma longa* extract containing a standardized amount of curcuminoids. The product was formulated in tablets, each one containing 500 mg of *Curcuma longa* (equivalent to 100 mg of curcuminoids). Metabolome analysis was performed to better understand the changes of 24-h urinary metabolome composition following the extract consumption. The analysis revealed that curcumin induced changes in urinary metabolites. In particular, metabolites related to fatty acid metabolism, involved in energy production, and compounds related to inflammation were detected, suggesting a key role of curcumin on the regulation of metabolic and anti-inflammatory pathways. Furthermore, changes of several microbial metabolites clearly revealed, although indirectly, intestinal absorption of curcuma constituents and gut microbiota metabolic activity, thus demonstrating an interaction between curcumin and gut microbiota.

### 4.1. Curcumin Favors Beneficial Bacterial Strains in Gut Microbiota

Recently, an increasing number of studies have suggested that gut dysbiosis is linked with many metabolic diseases, and curcumin seems to have beneficial effects on gut microbiota, favoring the growth of beneficial bacteria strains.

Indeed, Zhai et al. [99] explored the effect of curcumin on ochratoxin-induced liver oxidative injury in an animal model of liver disease. A total of 720 ducks were randomly assigned into four different groups: control, ochhratoxin, curcumin (ducks fed a diet with 400 mg/kg curcumin), and ochhratoxin plus curcumin and treated for 21 days. The ducks were provided with the different pelleted diets and ad libitum access to feed and water. The authors demonstrated that curcumin counteracted ochhratoxin-induced oxidative injury and lipid metabolism disruption. By 16S rRNA gene sequencing of gut microbiota it was shown that curcumin supplementation was also able to neutralize the decrease in butyric acid-producing bacteria induced by ochratoxin, and increased the richness and diversity of gut microbiota [99]. Thus, the authors hypothesized that curcumin could alleviate liver oxidative injury by modulating the gut microbiota.

Rats fed a high-fat diet show an altered hepatic metabolism accompanied by modified gut microbiota composition and increased intestinal permeability. In a nonalcoholic fatty liver disease (NAFLD) rat model induced by high-fat diet [20], rats were randomly divided into three groups fed standard diet, high-fat diet, or high-fat diet plus curcumin (200 mg/kg of curcumin by gastric gavage, daily for four weeks), respectively. The addition of curcumin to the diet significantly shifted the composition of the microbiota toward that of lean control rats fed a standard diet. In particular, curcumin was able to significantly counteract the high-fat-diet-induced abundance of several genera that have previously been associated to diabetes and inflammation, such as *Ruminococcus* [100]. Moreover, the treatment with curcumin succeeded to decrease thirty-six potentially harmful bacterial strains positively correlated with hepatic steatosis [20]. These data suggest that curcumin may have the gut microbiota as target in the treatment of liver steatosis induced by high-fat diet.

Other studies confirmed that oral curcumin administration was able to remarkably shift the ratio between beneficial and harmful bacteria in gut microbiota community in favor of beneficial bacteria strains, such as *Bifidobacteria*, *Lactobacilli*, and butyrate-producing bacteria, and reduces the abundance of the pathogenic ones, such as *Prevotellaceae, Coriobacterales, Enterobacteria*, and *Rikenellaceae*, often associated to the onset of systemic diseases [101,102,103]. In particular, Shen et al. [101] investigated the regulative effects of oral curcumin administration on the gut microbiota of C57BL/6 mice. After receiving daily curcumin gavage in a dose of 100 mg/kg body weight for 15 days, the gut microbial composition was significantly modified, affecting the abundance of several representative pathogenic families such as *Prevotellaceae, Bacteroidaceae*, and *Rikenellaceae*.

In APP/PS1 mice, a model of Alzheimer’s disease, it was found that curcumin administration improved the spatial learning and memory abilities, also reducing the amyloid plaque burden in the hippocampus [104]. Concomitantly, curcumin altered significantly the relative abundances of bacterial strains such as *Bacteroidaceae, Prevotellaceae*, and *Lactobacillaceae*, which have been reported to be key bacterial species associated with Alzheimer’s disease development [23]. In another study [105], estrogen deficiency induced in rats by ovariectomy gave rise to changes in the structure and distribution of intestinal microflora. Curcumin, administered at 100 mg/kg/day by oral gavage to ovariectomised rats, was able to partially reverse changes in the diversity of gut microbiota after 12 weeks of treatment [105].

It is important to underline that curcumin treatment decreases the microbial abundance of cancer-related species [106], such as *Prevotella* that were found to be greater in stool from CRC patients than in that from cancer-free patients [107]. Mice with colon cancer were fed different pelleted diets, with a calculated human equivalency dose of curcumin ranging from 8/mg/kg/day to 162 mg/kg/day [102]. Curcumin administration, at the highest dose, reduced or eliminated colon tumor burden, increasing *Lactobacilli* and reducing *Coriobacterales*. It has also been clearly demonstrated that curcumin treatment reduces several *Ruminococcus* species [108]; this represents an interesting finding because increased population of *Ruminococcus* species has been linked to CRC occurrence [90,109], even if the pathogenic role of *Ruminococcus* in cancer development has not been yet fully clarified. Moreover, in mice treated with a mutagenic compound, dietary curcumin was able to restore to control levels the amount of *Lactobacilli* [102], which have been shown to possess antitumoral function [110]. All these results support the potential anticancer activity of curcumin, at least against CRC, and have prompted researchers to start clinical trials focused to define this issue.

Peterson et al., in a human randomized placebo-controlled trial [108], investigated the effects of turmeric and curcumin dietary supplementation vs. placebo on 30 healthy subjects (10 for each group) previously advised not to consume any other curcumin-containing food or supplements for the entire study period. The turmeric tablets contained 1000 mg *Curcuma longa* plus 1.25 mg extract of piperine; the curcumin tablets contained 1000 mg curcumin plus 1.25 mg extract of piperine; the subjects were instructed to take three tablets orally with food, twice a day (total 6000 mg daily). Microbiota analyses were performed at baseline and after 8 weeks of treatment. All the subjects showed both significant variations of microbiota composition over the time and an individualized response to treatment. The intestinal microflora varied significantly from person to person, and the responses to the treatment were not uniform across individuals. However, comparing the number of bacterial species present in each group before and after the treatments, the placebo group showed an overall reduction in species by 15%, whereas the turmeric- and curcumin-treated groups displayed increases by 7% and 69%, respectively.

All these studies strongly suggest a protective effect of curcumin most likely based on its ability to promote an evident shift from pathogenic to beneficial bacteria strains in the gut.

However, it must be highlighted that these studies provide data hardly comparable because they use different doses and formulations. Moreover, the prebiotic effect of curcumin on gut microbiota is probably due to an indirect effect. Indeed, it is unlikely that curcumin metabolism provides a “direct” fitness advantage to any bacterial species: probably its prebiotic effect is the result of the induced host changes that in turn alter the gut microbiota.

In the light of the increasing data supporting a role of gut microbiota in the pathogenesis of many diseases, the research findings defining the ability of curcumin to positively modulate gut microbiota may help us to better understand its therapeutic benefits.

### 4.2. Curcumin Acts on Intestinal Barrier Function

Curcumin not only modifies the composition of the microbiota but might also enhance the function of the intestinal barrier. The intestinal barrier primarily is composed of four different layers. In the first one, the presence of alkaline phosphatase can detoxify bacterial endotoxin lipopolysaccharide. The second layer (mucosa) inhibits the entry of pathogenic bacteria. The third layer consists of tight junctions between intestinal epithelial cells, which form a barrier against bacterial endotoxin. Antibacterial proteins, which do not allow bacteria to cross the intestinal barrier, constitute the final layer [111]. Obviously, any defects in the intestinal barrier integrity can provoke an invasion of bacteria into normal colonic tissue, giving rise to a dysregulation of intestinal epithelial cells [112] and a subsequent local inflammation. Chronic inflammation underlies the development of western-induced metabolic diseases, such as diabetes or atherosclerosis, but it is also believed to be one of the primary reasons for the initiation of CRC.

In vitro studies have demonstrated that curcumin represents a potential compound to restore disrupted intestinal permeability. Indeed, in CaCo2 cells, curcumin is able to attenuate the disruption of intestinal epithelial barrier function, counteracting LPS-induced IL-1β secretion and preventing tight junction protein disruption [113,114]. Furthermore, curcumin was also able to decrease p38 MAPK activation, induced by IL-1β, and the subsequent raise in the phosphorylation of tight junction proteins and resulting disruption of their normal arrangement [114].

These results have been confirmed in animal models; in rats fed a high-fat diet for 16 weeks, curcumin treatment (200 mg/kg by daily oral gavage) improved the structure of intestinal tight junctions, also reducing serum concentrations of TNF-α, and LPS and upregulating the expression of occludin in the intestinal mucosa [115]. In a similar study, mice fed a Western diet for 16 weeks, and supplemented with curcumin (100 mg/kg by daily oral gavage), significantly improved intestinal barrier function, restoring the intestinal alkaline phosphatase activity and the expression of tight junction proteins ZO-1 and claudin-1 [116].

It is well known that the decreased expression of tight junction proteins, such as ZO-1 and occludin, plays a key role also in the pathophysiology of NAFLD [117]. Starting from this assumption, Feng et al. [20] demonstrated the beneficial effect of curcumin on the intestinal barrier integrity in NAFLD rats. Immunohistochemical data and western blot analysis showed that protein expression levels of ZO-1 and occludin were reduced in distal ileal tissues from NAFLD rats but were restored following curcumin administration (200 mg/kg of curcumin by gastric gavage, daily for four weeks). This work clearly highlighted that curcumin, by improving intestinal barrier integrity in vivo, might have a role in a novel approach addressed to NAFLD therapy.

All together, these results provide convincing evidence that curcumin contributes to the maintenance of intestinal barrier integrity, and thus may represent a new tool in preventive/therapeutic strategies against intestinal pathologies. As previously stated in the introduction, the studies included in this review were obtained using as keywords “curcumin and microbiome/microbiota”. This could represent a limitation of this section.

### 4.3. Curcumin Effects on Gut Inflammation

In a randomized placebo-controlled human trial, fifty-eight NAFLD patients were randomly allocated into two groups, which either received 250 mg curcumin–phospholipid delivery system, which was equivalent to 50 mg/day pure curcumin, or placebo [118]. Metabolomic analysis showed the beneficial effects of curcumin on biomarkers of oxidative stress and inflammation, which are considered two features of NAFLD. The authors suggested that curcumin treatment counteracted the increase of some bacterial strains that were changed during NAFLD progression.

An in vivo animal study [119] reported that a newly developed nanoparticle curcumin actively improves inflammation in mice with DSS (dextrane sulfate sodium)-colitis by inhibiting the expression of proinflammatory mediators and inducing Treg expansion which was also accompanied by the increase of fecal butyrate levels. Curcumin was mixed with the powder form of a normal rodent diet (containing 0.2% (*w*/*w*) nanoparticle curcumin): this compound was able to suppress the activation of NF-κB and the expression of proinflammatory mediators in the colonic epithelial cells of treated mice.

Alternatively, curcumin could act by attenuating LPS-induced inflammation by inhibiting the activation of TLR4/MyD88/NF-κB signaling pathways [120,121]. Moreover, curcumin has been shown to inhibit NF-κB nuclear translocation and to mitigate the expression of other pro-inflammatory genes that are overactivated in cancer [122].

In Weaned piglets fed a diet supplemented with curcumin (300 mg/kg of curcumin, mixed with the normal diet) for 28 days, Gan et al. demonstrated that this polyphenol was able to alleviate inflammation downregulating the expression of TLR4 by inhibiting *Escherichia coli* proliferation [123].

Unfortunately, only limited studies have been performed on human subjects up to now and, despite all the beneficial effects of curcumin described so far in in vivo studies, these results must be consistently supported through larger human clinical trials.

## 5. Gut Microbiota Metabolizes Curcumin

**Figure 2:**
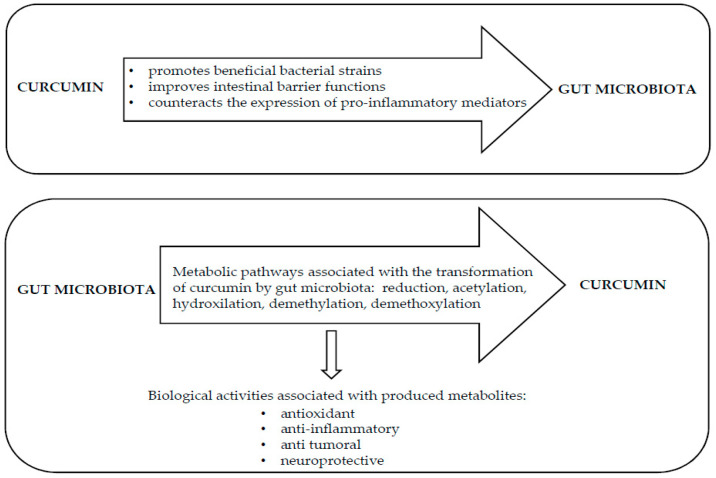
Reciprocal interaction between curcumin and gut microbiota.

It is worth noting that the interaction between curcumin and microbiota is bidirectional (Figure 2). Consequently, an important effect of gut microbiota on curcumin has been evidenced. The metabolic transformation of curcumin does not occur only in the enterocytes and in hepatocytes, but it is also carried out by enzymes produced by the gut microbiota that generate many active metabolites [44]. The biological activity of curcumin metabolites may differ from that of the native curcumin, and specific biological properties attributed to curcumin actually depend on bioactive metabolites produced by gut microbiota digestion [124].

Like for other dietary polyphenols such as anthocyanins, the biological activities of curcumin are related not only to the absorption rate, but also to the digestion by intestinal flora that leads to active curcumin metabolites. Moreover, the possible beneficial effects of curcumin depend not only on the dietary intake of curcumin, but also on the individual capacity of metabolizing it, that is, ultimately, on the composition of the gut microbiota of each person.

Several enteric bacteria capable of modifying curcumin have been identified: analyses of microorganisms isolated from human feces have previously shown that *Escherichia coli* represents the bacteria with the highest curcumin-metabolizing activity, via the NADPH-dependent curcumin/dihydrocurcumin reductase. This enzyme is able to convert curcumin into dihydrocurcumin, and then in the final product, tetrahydrocurcumin [125]. Other microorganisms such as *Bifidobacteria longum, Bifidobacteria pseudocatenulaum, Enterococcus faecalis, Lactobacillus acidophilus*, and *Lactobacillus casei* represent relevant bacterial strains able to metabolize curcumin, with a reduction of the parent compound higher than 50% [126].

Lou et al. [22] assessed whether curcumin was metabolized in vitro by human intestinal microorganisms, performing an ultra-performance liquid chromatography analysis, coupled with quadrupole time-of-flight mass. Curcumin (100 µM) was added to the intestinal bacteria culture obtained from fresh human fecal samples. The results clearly indicate that curcumin was extensively biotransformed by intestinal microflora, yielding 23 different metabolites also revealing different metabolic pathways, such as acetylation, hydroxylation, reduction, demethylation, or a combination of them, by which curcumin was metabolized by human intestinal microflora. The predominant metabolites produced by this intestinal microflora system derive from the reduced curcumin.

In addition to the known reductive metabolism of curcuminoids, alternative biotransformation pathways by human gut microbiota have been highlighted [127]. Fresh fecal samples from three healthy volunteers were taken for the preparation of mixed-cell cultures, and a curcuminoid mixture (10 mM), composed of curcumin, demethoxycurcumin, and bisdemethoxycurcumin, was added to begin the biotransformation. The human intestinal bacterium *Blautia* sp. (MRG-PMF1), through a demethylation process, was able to convert curcumin into two metabolites, bis-demethylcurcumin and demethylcurcumin [127]. Interestingly, the demethylated curcuminoid metabolites were present only in the culture where *Blautia* sp. was added, thus confirming that curcuminoids are differently metabolized depending on the individual fecal microflora.

In an in vitro model containing human fecal starters to investigate the colonic metabolism of curcuminoids [128], it was demonstrated that after 24 h of fermentation, up to 24% of curcumin, 61% of demethoxycurcumin, and 87% of bisdemethoxycurcumin were degraded by the human fecal microbiota. Three main metabolites were detected in the fermentation cultures: tetrahydrocurcumin, dihydroferulic acid, and 1-(4-hydroxy-3-methoxyphenyl)-2-propanol. There is evidence that curcumin metabolites have properties and potency similar to curcumin: tetrahydrocurcumin exhibits the same physiological and pharmacological properties of the parental compound, probably by means of the beta-diketone moiety, as well as phenolic hydroxyl groups [129]. Moreover, tetrahydrocurcumin is able to prevent oxidative stress and neuroinflammation, exhibiting also anticancer effects, probably due to inhibition of significant cytokine release, such as IL-6 and TNFα [130]. Consequently, the bacterial breakdown products should be considered in further studies on curcumin since they could be responsible for beneficial effects.

In transgenic mice with Alzheimer disease [23], the biotransformation of curcumin induced by gut microbiota was studied. The mice were divided in three groups (high-level curcumin group, low-level curcumin group, and control). Curcumin was administered daily at 200 mg/kg body weight (high) or 50 mg/kg (low) by oral gavage for 3 months, and the metabolites of curcumin from the feces of mice were identified by HPLC-Q-TOF/MS spectroscopy analysis. The authors shown that administered curcumin was transformed by gut microbiota through reduction, demethoxylation, demethylation, and hydroxylation processes. Eight metabolites of curcumin were identified (bisdemethylated hexahydrocurcumin, demethylated and dehydroxylated hexahydrocurcumin, demethylcurcumin, demethylated and demethoxylated curcumin, hydroxylatedcurcumin, dihydrocurcumin, hexahydrocurcumin, and demethylated hexahydrocurcumin). It is important to highlight that many of these metabolites have been reported to exhibit neuroprotective ability [131,132,133].

These findings not only could explain the paradox between the pharmacological effect of curcumin and its poor bioavailability, but also suggest that curcumin transformed by gut microbiota might act as an useful tool for microbiome-targeting therapies for Alzheimer disease [23].

Overall, all these results demonstrated that gut microbiota had a profound impact on the biotransformation of curcumin, also showing the huge potential of curcumin metabolites produced by the intestinal microflora as promising substances for the prevention or treatment of many diseases. The bidirectional interaction between curcumin and gut microbiota is reported in Figure 2.

These results clearly highlight how the different composition of the microbiota among the individuals will cause different biotransformation of dietary curcumin. Accordingly, the beneficial effects depend not only on the curcumin taken from the diet, but also on the type of microbial population of the individual. Therefore, future researches on human volunteers are needed to provide a basis for gut microbiota-based therapeutic applications of curcumin.

## 6. Conclusions and Perspectives

This review highlighted the strong connection between curcumin and gut microbiota with the final aim of adding novel insight for defining additional mechanism of action of curcumin. More knowledge available about the bidirectional interaction between gut microbiota and curcumin seems to clarify the paradox of the low-bioavailability curcumin and its wide impact on health.

However, in vivo human studies on this topic are almost lacking. Additional human trials, which also take into account an accurate dietary assessment to investigate better the relation between diet and microbiota, are needed. This could allow us to understand the complex interactions between gut microflora and curcumin, providing a better comprehension of its therapeutic efficacy.

We would expect that, in the near future, extensive research will allow to define the gut microbiota as a biomarker for many diseases and the use of curcumin and other probiotics as possible agents to treat dysbiosis and associated diseases. Considering that more than two-thirds of patients do not disclose supplement use to their health care providers nowadays [134], it would be relevant to reinforce the need to consume curcumin, like any other supplement, exclusively under the supervision of health care medical providers.
